# Endothelial Activation by Platelets from Sickle Cell Anemia Patients

**DOI:** 10.1371/journal.pone.0089012

**Published:** 2014-02-13

**Authors:** Renata Proença-Ferreira, Ana Flávia Brugnerotto, Vanessa Tonin Garrido, Venina Marcela Dominical, Daiana Morelli Vital, Marilene de Fátima Reis Ribeiro, Melissa Ercolin dos Santos, Fabíola Traina, Sara T. Olalla-Saad, Fernando Ferreira Costa, Nicola Conran

**Affiliations:** 1 INCT do Sangue, Haematology and Haemotherapy Centre, School of Medicine, University of Campinas – UNICAMP, Campinas, São Paulo, Brazil; 2 Children's Center for Hematologic Investigations, Dr. Domingos A. Boldrini, Campinas, Brazil; 3 Department of Internal Medicine, University of São Paulo at Riberão Preto Medical School, Riberão Preto, Brazil; Emory University/Georgia Insititute of Technology, United States of America

## Abstract

Sickle cell anemia (SCA) is associated with a hypercoagulable state. Increased platelet activation is reported in SCA and SCA platelets may present augmented adhesion to the vascular endothelium, potentially contributing to the vaso-occlusive process. We sought to observe the effects of platelets (PLTs) from healthy control (CON) individuals and SCA individuals on endothelial activation, *in vitro*. Human umbilical vein endothelial cells (HUVEC) were cultured, in the presence, or not, of washed PLTs from CON or steady-state SCA individuals. Supernatants were reserved for cytokine quantification, and endothelial adhesion molecules (EAM) were analyzed by flow cytometry; gene expressions of *ICAM1* and genes of the NF-κB pathway were analyzed by qPCR. SCA PLTs were found to be more inflammatory, displaying increased adhesive properties, an increased production of IL-1β and a tendency towards elevated expressions of P-selectin and activated α_IIb_β_3_. Following culture in the presence of SCA PLTs, HUVEC presented significant augmentations in the expressions of the EAM, ICAM-1 and E-selectin, as well as increased IL-8 production and increased *ICAM1* and *NFKB1* (encodes p50 subunit of NF-κB) gene expressions. Interestingly, transwell inserts abolished the effects of SCA PLTs on EAM expression. Furthermore, an inhibitor of the NF-κB pathway, BAY 11-7082, also prevented the induction of EAM expression on the HUVEC surface by SCA PLTs. In conclusion, we find further evidence to indicate that platelets circulate in an activated state in sickle cell disease and are capable of stimulating endothelial cell activation. This effect appears to be mediated by direct contact, or even adhesion, between the platelets and endothelial cells and via NFκB-dependent signaling. As such, activated platelets in SCD may contribute to endothelial activation and, therefore, to the vaso-occlusive process. Results provide further evidence to support the use of anti-platelet approaches in association with other therapies for SCD.

## Introduction

Sickle cell anemia is a genetic disease caused by the production of abnormal hemoglobin S (HbS), which polymerizes under hypoxic conditions, resulting in the formation of sickled red blood cells that are less flexible and are liable to lysis. These alterations cause vaso-occlusive processes and hemolytic events that cause irreversible damage to organs and manifestations, such as painful vaso-occlusive crises, acute chest syndrome, stroke, osteonecrosis, leg ulcers and cardiovascular disease [1]. The vaso-occlusive process is the consequence of a complex pathophysiology that involves chronic vascular inflammation, hypoxia-reperfusion processes, oxidative stress and reduced nitric oxide bioavailability with ensuing endothelial activation and the adhesion of red and white cells to the vascular wall, leading to compromised blood flow of the small and microcirculatory blood vessels [2].

Thrombotic complications, including ischemic stroke, can occur in the sickle cell diseases (SCD) [3] and platelet activation and a hypercoagulable state are now thought to contribute to SCD pathophysiology [4]. Activation of the coagulation system and augmented thrombin generation in SCD [5] is indicated by reports of increased plasma levels of prothrombin fragment 1.2 (F1.2), thrombin anti-thrombin (TAT) [6], [7] and D-dimer levels [8], as well as augmented tissue factor (TF) expression in patients [9]–[11]. Platelets of SCA patients (SCA platelets) are also known to circulate in an activated state [12]–[14], presenting altered aggregation [15], [16] and increased adhesive properties under static conditions [14]. SCA platelets are reported to present an increased expression of adhesion molecules and markers of platelet activation, such as CD40 ligand (CD40L), on their surface [14], [17], [18] and produce higher levels of potent inflammatory cytokines, such as TNFSF14 (Tumor necrosis factor ligand superfamily member 14; LIGHT; CD258) [19]. Furthermore, increased circulating levels of platelet microparticles and platelet-derived proteins, such as thrombospondin-1 (TSP-1) and platelet factor 4 (PF4), are a further indication of platelet activation in SCA [13], [20]–[22].

The exact mechanism by which platelets may be activated in SCD is not clear, but the release of adenosine diphosphate (ADP) from lysed red blood cells may contribute to platelet activation [23], and the exposure of phosphatidyl serine (PS) on the surface of sickle red blood cells is also suggested to activate platelets, via induction of thrombin generation [24], [25] and a consequent reduction in intraplatelet cAMP (cyclic adenosine monophosphate) [14]. Low nitric oxide (NO) bioavailability may also activate platelets in SCD [2]. Additionally, SCD platelets can form circulating heterocellular aggregates with monocytes and neutrophils, where the adhesion of platelets to these leukocytes is suggested to participate in their activation and subsequent adhesion to the endothelium [26], [27]. Activated α_IIb_β_3_ expression has also been previously correlated to the severity of pulmonary hypertension in SCD and to laboratory markers of hemolysis, such as reticulocyte counts [28].

While a role for the platelet in SCD pathophysiology seems apparent, whether the platelets have a direct role in the vaso-oclusive process is not clear. We hypothesize that the close proximity or adhesion of activated platelets to the vascular endothelium may contribute to the activation of endothelium in SCD, an event that is central to the vaso-occlusive process. We sought to observe the effects of platelets from healthy individuals and SCA individuals on the expression and production of adhesion molecules and inflammatory proteins by endothelial cells *in vitro*, and to investigate the signaling pathway that may mediate this effect.

## Patients and Methods

### Materials

Human fibrinogen and BAY 11-7082 ((E)3-[(4-Methylphenyl)sulfonyl]-2- propenenitrile) were purchased from Calbiochem (La Jolla, CA, USA); Ham's F12K medium was from Gibco-Invitrogen (Carlsbad, CA, USA). Collagen type I, fetal bovine serum and trypsin/ethylenediaminetetraacetic acid (EDTA) solution were obtained from Sigma-Aldrich (Saint Louis, MO, USA). Recombinant TNF-α and ELISAs for interleukin-8 (IL-8), interleukin-1β (IL-1β) and Platelet Factor 4 (PF4) determination were from R&D Systems (Minneapolis, MN, USA). ACD Vacutainers® were used for blood collection (BD Brasil, Sao Paulo, Brazil). All other products were from Sigma-Aldrich unless otherwise stated.

### Patients

A total of 90 steady-state SCA patients (between 18-53 years old) were recruited from the Hematology and Hemotherapy Center, University of Campinas, Brazil and from the Boldrini Children's Center for Hematologic Investigations, Campinas, Brazil. All patients were diagnosed as homozygous for HbS (using hemoglobin electrophoresis methods and high performance liquid chromatography). All patients attended regular clinics and had not experienced painful crisis nor received blood transfusions in the preceding 3 months and had not taken aspirin during the previous 10 days. Patients on hydroxyurea (HU) therapy had been taking 15–30 mg/kg per day for at least 3 months. A total of 55 healthy volunteers (between 19–61 years old) were recruited at the same centers. None of the controls had taken any medication within the last 10 days and controls were age- and gender-matched where possible. See Table 1 for clinical details of participating patients and healthy controls.

**Table 1:** Demographic, clinical and hematological details of all controls and patients participating in the study.

	CONTROLS	SCA	p
**Male/female**	18/37	46/44	
**Age (years)**	34.1 (31; 24, 58)	34.3 (32; 18; 53)	
**Hydroxyurea therapy (n)**	-	56	
**Red blood cell count (10^6^/µL)**	4.70 (4.64; 3.79; 5.60)	2.61 (2.61; 1.63; 4.78)	**p<0.001**
**Hematocrit (%)**	39.9 (39.5; 32.1; 48.4)	24.8 (22.6; 17.; 37.4)	**p<0.001**
**Hemoglobin (g/dL)**	13.5 (13.3; 10.9; 15.6)	8.5 (8.0; 6.0; 12.9)	**p<0.001**
**Mean corpuscular volume (fL)**	84.6 (84.3; 74.9; 95.7)	96.1 (93.8; 69.2; 126.6)	**p<0.001**
**Mean corpuscular hemoglobin (pg)**	28.8 (29.0; 25.2; 30.8)	33.4 (33.1; 20.9; 47.0)	**p<0.01**
**WBC (10^3^/µL)**	6.1 (5.90; 3.6; 8.9)	9.6 (9.5; 4.0; 22.3)	**p<0.001**
**HbF (%)**	0.4 (0.3; 0.1; 1.7)	10.6 (8.7; 0.8; 27.2)	**p<0.001**
**PLTs (10^3/^µL)**	293 (276; 208; 428)	375 (355; 156; 711)	**p<0.01**

SCA, steady-state SCA patients; hydroxyurea therapy (20–30 mg/kg/day for at least 3 months); HbF, fetal hemoglobin. Data present (except M/F value) are mean (median, min, max).

### Ethics Statement

Informed written consent was obtained from all patients and controls and the study was approved by the Ethics Committees of the University of Campinas, Brazil and of the Boldrini Children's Center, Campinas, Brazil (Reg. no. 1170/2009). All clinical investigations were conducted in accordance with the Declaration of Helsinki.

### Preparation of washed platelets

Platelet rich plasma (PRP) was obtained by centrifugation of whole blood (collected in 22.0 g/L trisodium citrate, 8.0 g/L citric acid, 24.5 g/L dextrose) at 200 g, 21°C for 20 min. PRP was washed once in wash buffer (140 mmol/l NaCl, 5.0 mmol/l KCl, 12 mmol/l sodium citrate, 10 mmol/l glucose and 12.5 mmol/l sacarose, pH 6) and platelets (PLTs) were isolated from the PRP by centrifugation at 800 g at 21°C for 12 min. The platelet pellet was then resuspended in F-12K medium (GIBCO *life* Technologies, Grand Island, NY, USA). Platelet counts were performed using a Beckman Coulter cell counter (Fullerton, CA, USA). Suspensions were utilized immediately in assays; the anticoagulant utilized did not affect assay results. Control and SCD platelets were treated in the same manner and always run in parallel in platelet assays; thus, differences in properties of platelets reflect differences inherent to these platelets, and are not due to the separation/washing procedure.

### Microfluidic platelet adhesion assay

The Venaflux™ microfluidic platform (Cellix Ltd, Dublin, Ireland) was used to measure platelet adhesion to fibrinogen (FB, 50 µg/mL) and collagen (Col, 100 µg/mL) under physiologically relevant conditions. Unwashed PRP was obtained from control individuals and/or SCA patients and resuspended in phosphate buffered saline (PBS, pH = 7.4; 1×10^7^ PLTs/mL) to monitor adhesion to biochips (400 µm-wide Vena 8 microchannels, Cellix, Dublin, Ireland) that were pre-coated (2 h, room temperature) with FB or Col and subsequently blocked with 1% BSA/PBS. Platelets were perfused over micro channels at a shear stress of 0.3 dyne/cm^2^ for 3 min at 37°C; adhesion was detected using brightfield inverted microscopy and images were acquired with a DeltaPix camera (Carl Zeiss Inc, Jena, Germany; 40× magnification; DeltaPix Camera, Maalov, Denmark). The adhesion of platelets to duplicate microchannels was recorded at three positions in the channel and analyzed using the DucoCell analysis program (Cellix Ltd), recording the mean number of platelets adhered to an area of 330×250 µm (0.08 mm^2^).

### Culture of endothelial cells

Human Umbilical Vein Endothelial Cells (HUVEC) were purchased from the American Type Culture Collection (ATCC) (Manassas, VA, USA) and cultured in 75 cm^2^ flasks, and 6-well tissue culture plates with Ham's 12K medium supplemented with endothelial cell growth supplement factor (ECGS; 0.5 mg/mL), heparin (0.1 mg/mL) and fetal bovine serum (10%). Cells were used at the fourth to sixth passage and cultures were maintained at 37°C in a humidified atmosphere with 5% CO_2_; the medium was replaced every 2 days until confluence (3–5 days).

### *In vitro* co-culture assay

HUVEC were co-cultured (1×10^6^ cells/well, 4 h, 37°C, 5% CO_2_) in the presence or absence of PLTs (1×10^8^ PLTs/well). In some cultures, transwell inserts (0.4 µm pores) were used to separate PLTs from HUVEC. For some experiments, HUVEC were also incubated with TNF-α (10 ng/mL in medium) (3 h, 37°C, 5% CO_2_) as a positive endothelial activation control. For other experiments, HUVEC were pretreated with BAY 11-7082 (20 µM) or vehicle (0.02% dimethylsulfoxide) for 30 min (37°C, 5% CO_2_) before performance of the co-culture assays. After incubation, PLTs were removed and the supernatants were stored (−80°C) for cytokine quantification by ELISA. HUVECs were washed once with PBS and detached with trypsin/EDTA solution (4 min, 37°C) for flow cytometry or quantitative PCR (qPCR).

### Flow Cytometry

After incubations, the PLTs were removed from cultures; HUVECs were washed once with PBS and detached with trypsin/EDTA solution trypsin (0.25%). Antibodies used for flow cytometry to recognize endothelial adhesion molecules were all purchased from BD Pharmingen (San Diego, CA, USA); anti-CD62P-fluorescein isothiocyanate (FITC; P-selectin; clone AK-4), PAC-1-FITC (recognizes α_IIb_β_3_ in its activated conformation), anti-CD54-phycoerythin (PE; ICAM-1, clone LB-2), anti-CD106-FITC (VCAM-1, clone 5110C9) and anti-CD62E-Allophycocyanin (APC; E-selectin, clone 68-5H11). Flow cytometric analysis (10 000 events) was performed using a FACSCalibur flow cytometer and BD FACS DIVA 7.0 software (BD, San Jose, CA, USA). Possible alterations in HUVEC phenotype and activation state following trypsin treatment should not be overlooked; however control and experimental HUVEC were treated with trypsin in a similar manner and differences between groups were therefore not the consequence of trypsin application.

### Quantification of IL-8, IL-1β and PF4 in culture supernatants

Levels of IL-8, IL-1β and PF4 were determined in the supernatants of platelets cultured for 4 h (1×10^8^ PLTs/well; 37°C, 5% CO_2_) and of co-cultures (4 h, 37°C, 5% CO_2_) of HUVEC (1×10^6^ cels/well) with platelets (1×10^8^ PLTs/well) using commercial high-sensitivity ELISAs, according to the manufacturer's instructions (R&D Systems).

### Quantification of endothelial gene expressions of *ICAM1*, *NFKB1*, *RELA*, *CHUK* and *IKKBETA* by qPCR

Extraction of mRNA from HUVEC (1–5×10^5^ cells) was performed with the RNeasey® Micro Kit (QIAGEN Biotecnologia Brasil Ltda, São Paulo, Brazil) and cDNA synthesized using a reverse-transcription kit (Thermo Scientific, Waltham, MA, USA). Synthetic oligonucleotide primers were designed to amplify cDNA for conserved regions of the *ICAM1* (encodes Intercellular Adhesion Molecule 1 protein), *NFKB1* (encodes the p50 subunit of nuclear factor-κB [NF-κB]), *RELA* (encodes the p65 subunit of NF-κB), *CHUK* (encodes the IκBα inhibitor protein for NF-κB) and *IKKBETA* (encodes the I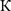 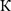
β kinase protein for NF-κB) genes by Primer-Express (Applied Biosystems, Foster City, CA, USA). For primer sequences, see Table 2. Primers were synthesized by Invitrogen (São Paulo, Brazil) and *ACTB* and *GAPDH* were used as internal control genes. All samples were assayed in a 12 µL volume containing 5 ng cDNA, 6 µL SYBR Green Master Mix PCR (Applied Biosystems, Foster City, California, USA) and gene primers (7500 Fast Real-Time PCR System – Applied Biosystems). To confirm accuracy and reproducibility of real-time PCR, the intra-assay precision was calculated according to the equation: E^(-1/slope)^. The dissociation protocol was performed at the end of each run to check for non-specific amplification. Two replicas were run on the plate for each sample. Results are expressed as arbitrary units (A.U.) of gene expression, when compared with the control genes.

**Table 2:** Primer sequences for performance of real-time quantitative PCR.

Genes	Primers	Optimal concentration (nM)
*ICAM1 - F*	5′-GGAAATACTGAAACTTGCTGCCTAT-3′	150
*ICAM1 - R*	5′-ACACATGTCTATGGAGGGCCAC-3′	
*NFKB1 - F*	5′-GCTTTTGGTGTCCTTGGGT-3′	70
*NFKB1 - R*	5′-AGGTCCACTGCGAGGTGA-3′	
*RELA - F*	5′-CACCTCGACGCATTGCTGT-3′	70
*RELA - R*	5′-GACGTAAAGGGATAGGGCTGG-3′	
*CHUK - F*	5′-ACCAGCATCGGGAACTTGATC-3′	70
*CHUK - R*	5′-TGGCACCATCGTTCTCTGTTT-3′	
*IKKBETA- F*	5′-ATCTCCGGAAGTACCTGAACCA-3′	70
*IKKBETA- R*	5′-AGCGCAGAGGCAATGTCACT-3′	
*ACTB - F*	5′-AAAGAGATGGCCACGGCTGCT-3′	150
*ACTB - R*	5′-TCGCTCCAACCGACTGCTGT-3′	
*GAPDH - F*	5′-GCACCGTCAAGGCTGAGAAC-3′	150
*GAPDH - R*	5′-CCACTTGATTTTGGAGGGATCT-3′	

*ACTB*, encodes β-actin protein; *CHUK*, encodes IκBα protein; *GAPDH*, encodes glyceradehyde 3-phosphate dehydrogenase; *ICAM1*, encodes ICAM-1 protein; *IKKBETA*, encodes IKKβ protein; *NFKB1*, encodes p50 protein; *RELA*, encodes p65 protein.

### Statistical analysis

Data are depicted as median values and groups were compared by unpaired nonparametric analysis of variance (ANOVA) followed by Kruskal-Wallis Comparisons test, or the unpaired nonparametric Mann-Whitney test for comparisons between two groups. P<0.05 was considered to be significant.

## Results

### Platelets from SCA patients demonstrate altered expressions and productions of adhesion molecules and inflammatory proteins

**Figure 1:**
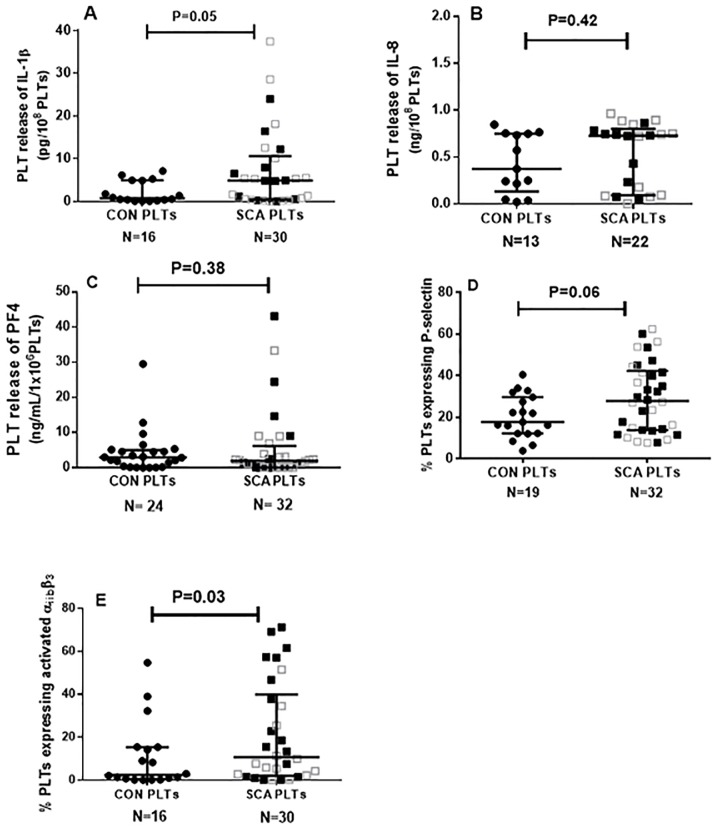
Platelets as inflammatory cells in SCA. Release of (A) IL-1β, (B) IL-8, (C) platelet factor 4 (PF4) from platelets of healthy control individuals (CON) and SCA patients in steady state (SCA). Cytokine release was determined by ELISA in platelet suspensions (1×10^8^ PLT/ml) after incubation for 4 h (37°C, 5% CO_2_). Expressions of (D) P-selectin and (E) activated α_IIb_β_3_ integrin on the surface of platelets from healthy control individuals (CON) and SCA patients in steady state (SCA). Medians and interquartile ranges are depicted. Adhesion molecule expression was determined by flow cytometry. Some of the data included in the data sets depicted in graphs 1D and 1E have been previously published in table form [14]. Closed black square symbols represent SCA patients not on HU therapy; open grey square symbols represent patients on HU (15–30 mg/kg/day) therapy.

Platelets of SCA individuals are known to circulate in a partially-activated state. Accordingly, platelets from individuals of our SCA population were found to express a number of inflammatory molecules (Figure 1). Platelets from healthy control individuals and SCA individuals (CON PLTs and SCA PLTs, respectively) were incubated in culture medium for 4 h at 37°C and the productions of the cytokines, IL-1β, platelet factor 4 (PF4) and IL-8 (Figure 1A–C) were determined by ELISA. While no significant alterations in the release of IL-8 or PF4 by SCA PLTs were observed (Fig. 1B, Fig. 1C), compared to control PLTs, IL-1β release by SCA PLTs was significantly increased for SCA PLTs. Flow cytometric analysis found the expression of the adhesion molecule, P-selectin (Figure 1D), to be increased on SCA PLTs (P = 0.06), but not quite significantly, while the α_IIb_β_3_ integrin was found in a more activated state on SCA PLTs (Figure 1E), as demonstrated by increased PAC-1 antibody binding. When PLT data were stratified into those PLTs that were from SCA patients on HU therapy and those that were not, no significant differences were found between any of these parameters for these two patient groups (P>0.05), with the exception of the expression of activated α_IIb_β_3_ integrin, which was found to be higher on PLTs from patients not on HU (30.2±6.6%, n = 16), compared to those on HU (11.6±4.1, n = 14; P = 0.05).

### SCA platelets demonstrate alterations in adhesive properties in a microfluidic assay

**Figure 2:**
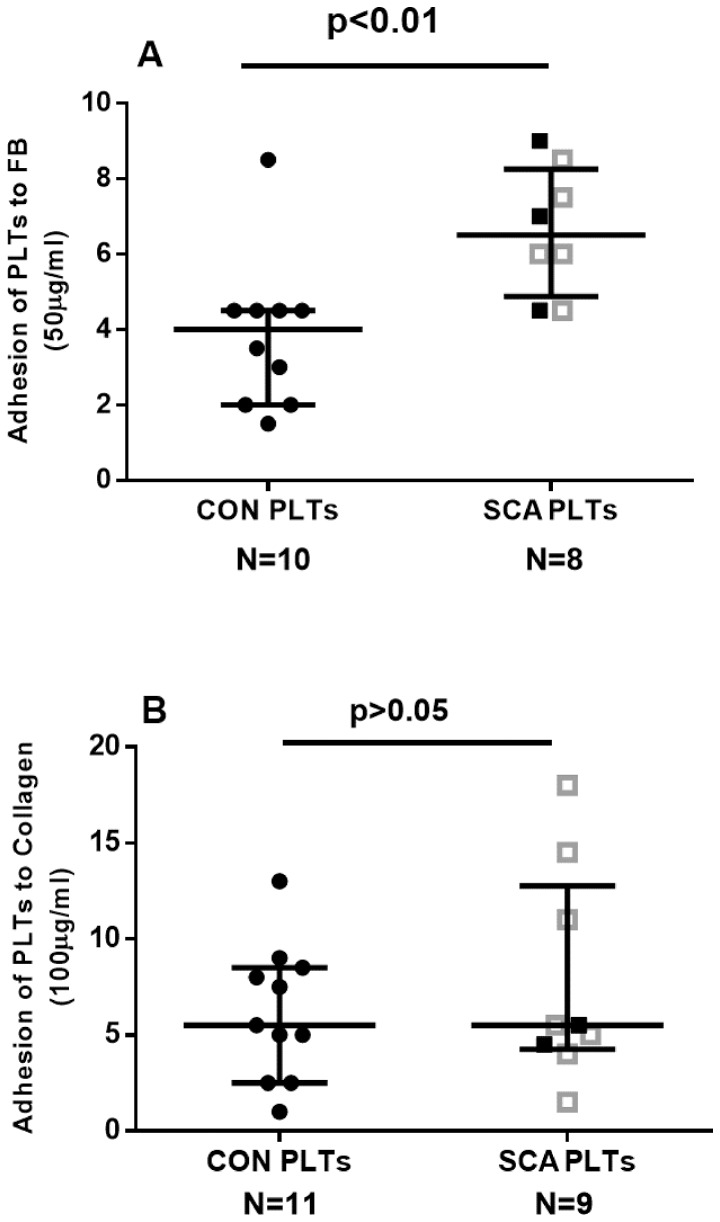
Adhesive properties of platelets. A microfluidic assay was used to determine the adhesion of platelets from healthy control individuals (CON) and SCA patients in steady state (SCA) to (A) 50 µg/ml fibrinogen and (B) 100 µg/ml collagen. Platelets (1×10^7^ PLTs/mL) were perfused over microchannels (400 µm width) coated with immobilized ligands at a shear stress of 0.3 dyne/cm^2^ for 3 min at 37°C; adhesion was detected using brightfield inverted microscopy. The adhesion of platelets to duplicate microchannels was recorded at three positions in each channel and analyzed using the DucoCell analysis program, recording the mean number of platelets adhered to an area of 0.08 mm^2^. Medians and interquartile ranges are depicted. Closed black square symbols represent SCA patients not on HU therapy; open grey square symbols represent patients on HU (15–30 mg/kg/day) therapy.

We have previously shown that platelets demonstrate an augmented capacity to adhere to fibrinogen ligand under static assay conditions [14], [29]. In accordance with these previous findings, using a microfluidic platform, SCA PLTs were found to adhere significantly more, under flow conditions, to microchannels coated with fibrinogen (See Figure 2A), but not to collagen (Figure 2B). No significant differences were found between the ability of platelets from SCA individuals and SCA patients on HU to adhere to fibrinogen or collagen (P>0.05), although the low experimental number employed for the SCA group may have limited the detection of any possible differences in adhesive properties.

### SCA platelets induce the expression of the adhesion molecules, ICAM-1 and E-selectin, on the surface of HUVEC

**Figure 3:**
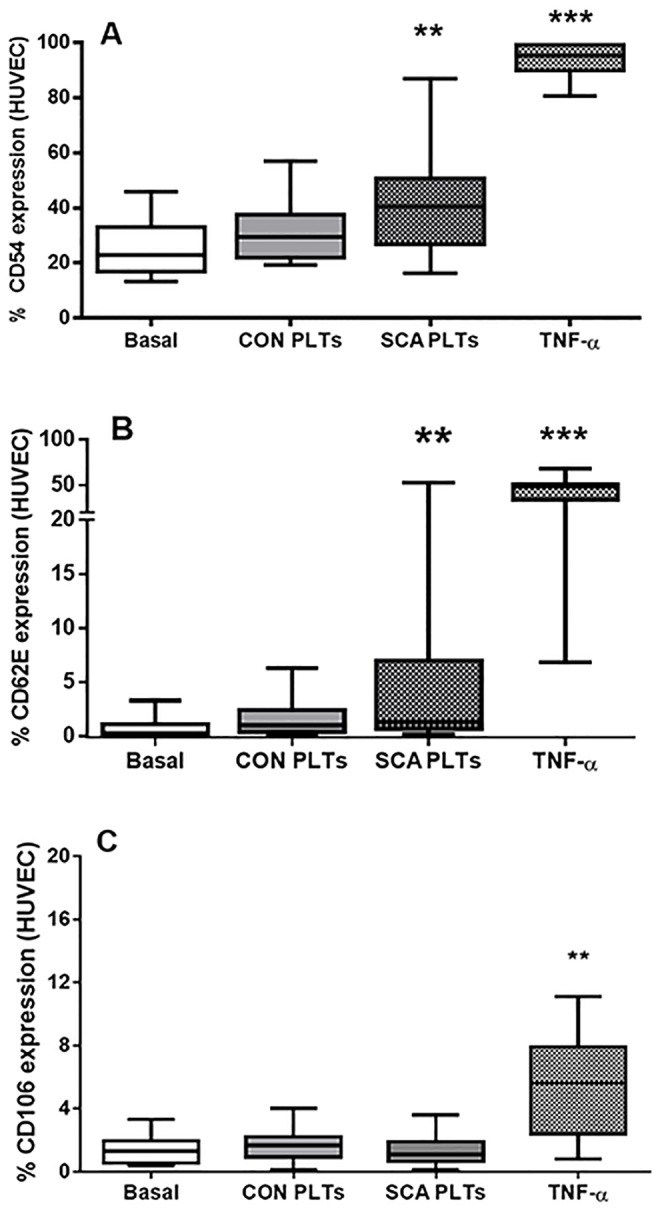
Effects of platelets on endothelial adhesion molecule expression. Surface expressions of (A) ICAM-1 (CD54), (B) E-selectin (CD62E) and (C) VCAM-1 (CD106) adhesion molecules on HUVEC following co-culture in direct contact with healthy control (CON; N≥15) PLTs, steady-state sickle cell anemia (SCA; N≥25) PLTs or TNF-α (N≥11). HUVEC (1×10^6^ cells/well) were incubated with CON or SCA PLTs (1×10^8^ PLTs/well) for 4 h (37°C, 5% CO_2_), or with TNF-α, (10 ng/ml; 3 h, 37°C, 5% CO_2_). Expression of ICAM-1, E-selectin or VCAM-1 was analyzed on HUVEC (10 000 cells) by flow cytometry using anti-CD54-PE, CD62E-APC and CD106-FITC. **, p<0.01; ***, p<0.001, compared to basal (N≥16).

Flow cytometry was utilized to compare the surface expressions of CD54 (ICAM-1), CD62E (E-selectin) and CD106 (VCAM-1) on HUVEC following their incubation, or not, with platelets. Confluent HUVEC were co-cultured in direct contact with platelets (1×10^8^ PLTs/well) from control individuals (CON PLTs), SCA patients (SCA PLTs) or culture medium for 4 h (37°C, 5% CO_2_). HUVEC cultured with CON PLTs demonstrated a non-significant increase in surface ICAM-1 and E-selectin (FIGURE 3A and 3B); in contrast, SCA PLTs induced a significant increase in both ICAM-1 and E-selectin on HUVEC (FIGURE 3A and 3B), while VCAM-1 expression was not induced by either type of PLT (FIGURE 3C). A TNF-α-inflammatory stimulus (10 ng/mL) was used as a positive control to observe the effect of a strong inflammatory stimulus on adhesion molecule expression on HUVEC (FIGURE 3).

PLTs from SCA patients on HU (SCAHU) therapy and from those patients not on HU demonstrated similar effects on HUVEC adhesion molecule expression, with no significant differences between the two subgroups. Mean CD54 (ICAM-1) expression on HUVEC following incubation (4 h, 37×C) with SCA and SCAHU platelets was 43.4±3.2% and 38.8±4.00%, respectively (n = 15 and 19); Mean CD62E (E-selectin) expression on HUVEC following incubation (4 h, 37°C) with SCA and SCAHU platelets; 2.91±0.93% and 7.78±3.44%, respectively (n = 15 and 16); Mean CD106 (VCAM-1) expression on HUVEC following incubation (4 h, 37°C) with SCA and SCAHU platelets; 1.44±0.24% and 1.16±0.21%, respectively (n = 17 and 8) (P>0.05 for all comparisons). As such, all results for both SCA and SCAHU individuals are depicted together in the figures for the HUVEC experiments and data were analyzed together in the same group (SCA).

### Transwell inserts abolish the effects of the induction of endothelial adhesion molecule expression by platelets

**Figure 4:**
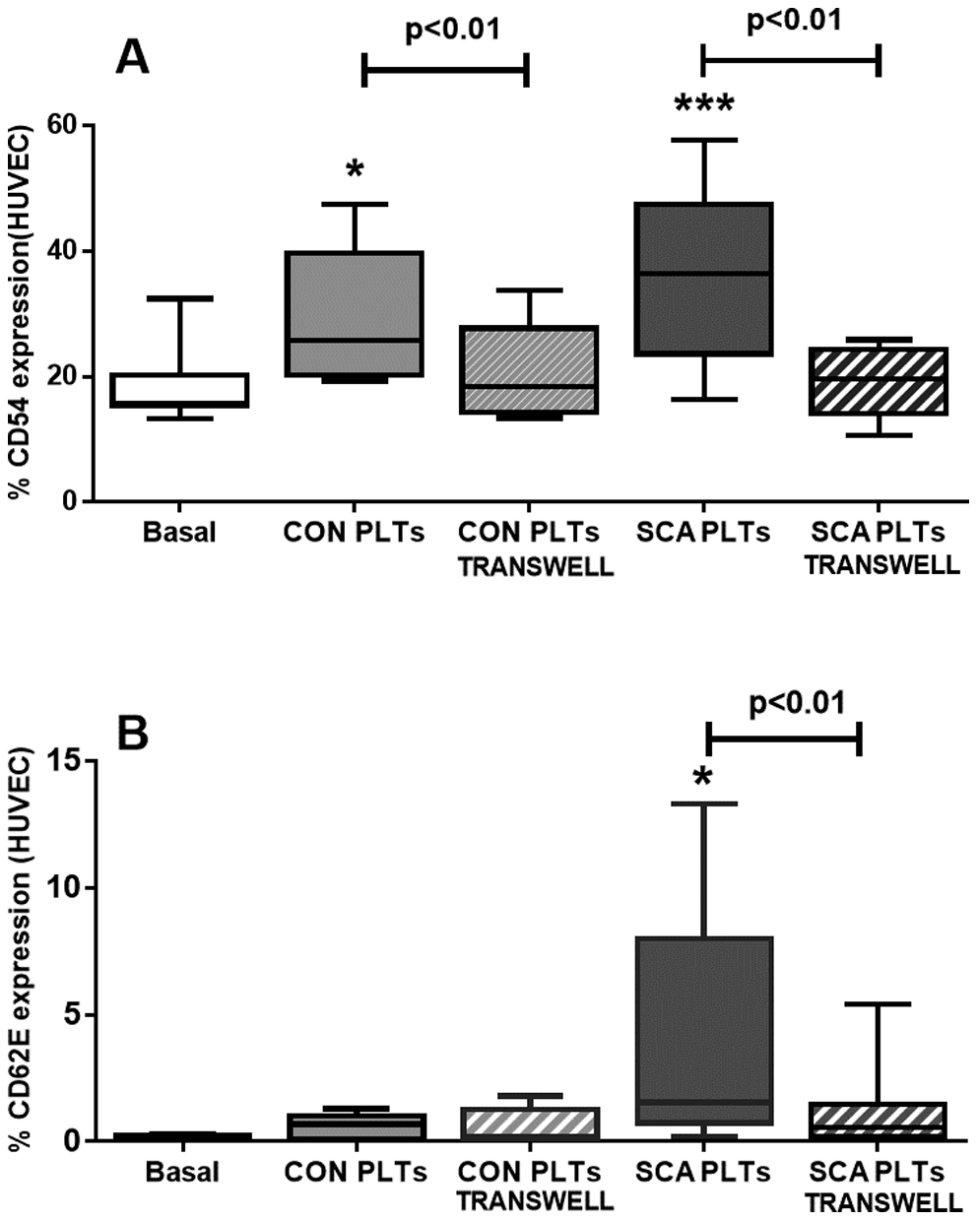
Transwell inserts reverse the effects of SCA platelets on endothelial adhesion molecules expression. Surface expressions of (A) ICAM-1 (CD54) and (B) E-selectin (CD62E) adhesion molecules on HUVEC (1×10^6^ cells/well) following culture in the presence of healthy control (CON; N≥6; 1×10^8^ PLTs/well) PLTs or steady-state sickle cell anemia (SCA; N ≥12; 1×10^8^ PLTs/well) PLTs and in the presence and absence of transwell inserts (0.4 µm pores). Expressions of ICAM-1 and E-selectin were evaluated by flow cytometry using anti-CD54-PE and CD62E-APC. *, p<0.01; ***, p<0.001, compared to basal (N = 9).

Repetition of these assays, but with the placement of transwell polystyrene membrane inserts (0.4 µm pores) in culture plates, to physically separate the PLTs from HUVEC, completely abolished the effects of CON and SCA PLTs on ICAM-1 (CD54) expression (FIGURE 4A) and the effect of SCA platelets on E-selectin (CD62E) expression (Figure 4B). Data indicate that close contact, or possibly adhesion, of the platelets with the HUVEC is required for activation of endothelial adhesion molecule expression.

### SCA PLTs increase the production of interleukin-8 (IL-8) by endothelial cells

**Figure 5:**
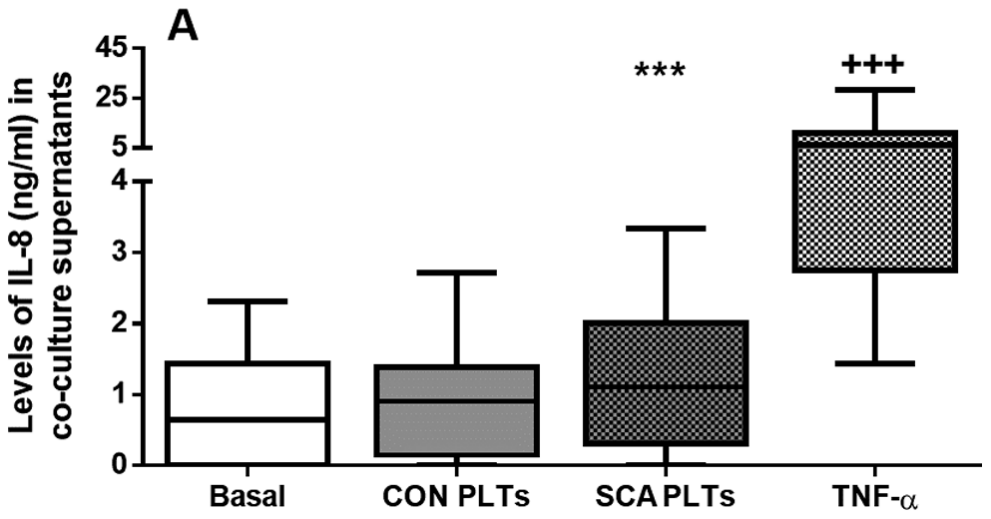
Release of IL-8 from co-cultures of HUVEC and PLTs. Levels of IL-8 (ng/ml) were quantified in supernatants of co-cultures of HUVEC (1×10^6^ cells/well) (4 h in suspension, 37°C, 5% CO_2_) in direct contact with CON (n = 27) or SCA PLTs (1×10^8^ PLTs/well; n = 44), (4 h, 37°C, 5% CO_2_); or following pre-incubation of HUVEC with TNF-α (10 ng/ml; N = 24; 3 h, 37°C, 5% CO_2_). ***, P<0.001, compared to basal (N = 24); +++, P<0.001, compared to all groups.

IL-8 is an important inflammatory cytokine and can be produced and released by endothelial cells under basal conditions. Production of IL-8 was significantly augmented in HUVEC cultures that were co-cultured in the presence of SCA PLTs, compared to basal levels of production by non-stimulated HUVEC (FIGURE 5). CON PLTs had no significant effect on IL-8 levels in HUVEC cultures, while TNF-α significantly increased IL-8 secretion by HUVEC. Platelets also produce and release IL-8, but at low levels when compared to IL-8 production by HUVEC (See Figure 1B); thus it seems likely that the IL-8 observed in these cultures may be produced primarily by stimulated HUVEC.

### Co-culture of HUVEC with SCA platelets induces gene expression of endothelial *ICAM1* and genes of the NF-κB pathway

**Figure 6:**
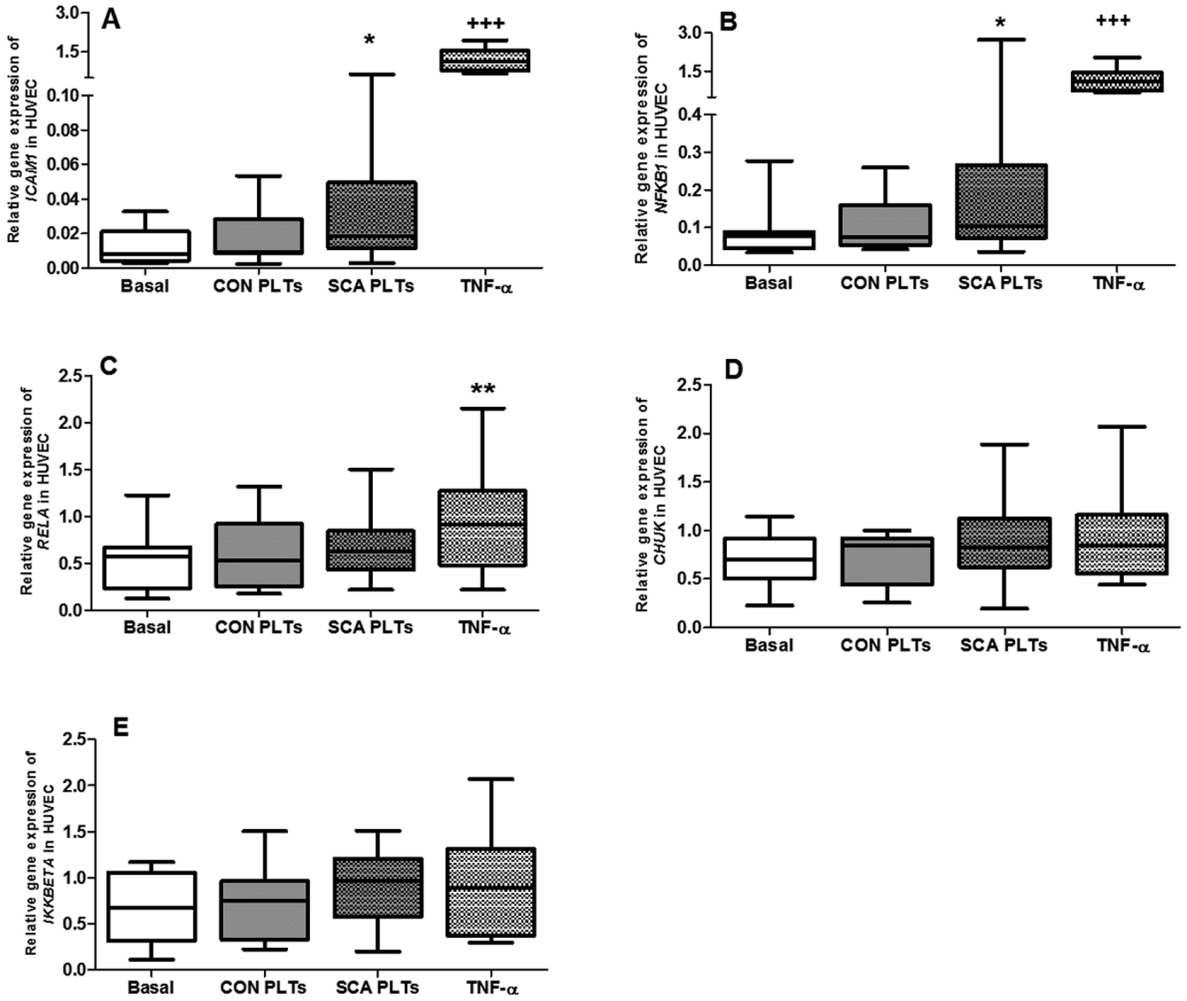
Effect of SCA platelets on endothelial gene expression. Relative gene expression of (A) *ICAM1*, (B) *NFKB1*, (C) *RELA*, (D) *CHUK* and (E) *IKKBETA* in HUVEC after co-culture with PLTs. HUVEC (1×10^6^ cells/well) were co-cultured in direct contact with CON (N = 10) or SCA PLTs (N≥24; 1×10^8^ PLTs/well) (4 h, 37°C, 5% CO_2_); or pre-incubated with HUVEC with TNF-α (10 ng/ml; N≥11; 3 h, 37°C, 5% CO_2_). Relative gene expressions were determined by qPCR and expressed relative to *ACTB* and *GAPDH* expressions. *, P<0.05; **,P<0.01, compared to basal (N≥11). +++, P<0.001, compared to all groups.

The effect of co-culture of PLTs with HUVEC on endothelial gene expression was determined by quantitative real-time PCR (qPCR). Confluent HUVEC were co-cultured in direct contact with platelets from control individuals (CON PLTs), SCA patients (SCA PLTs) or culture medium for 4 h (37°C, 5% CO_2_); PLTs were removed by gentle washing and HUVEC gene expression determined. Relative gene expression of *ICAM1* increased by 6.9-fold in endothelial cells in the presence of SCA PLTs, compared to basal expression, but was not altered in the presence of CON PLTs (FIGURE 6A). In contrast, TNF-α increased the *ICAM1* gene expression in HUVEC by 93-fold, when compared to basal expression (FIGURE 6A). The nuclear transcription factor, NF-κB, is a cytoplasmic protein that when activated translocates to the nucleus, under the control of proteins of the NF-κB pathway, inducing the gene expression of a number of genes, including inflammatory molecules, growth factors, and cell adhesion molecules [30], [31]. Following the incubation of HUVEC with SCA PLTs, but not CON PLTs, the expression of the *NFKB1* gene (encodes p50 subunit of NF-κB) was significantly increased in HUVEC (Figure 6B). In contrast, the expressions of the *CHUK* gene (encodes IκBα inhibitor protein), the *IKKBETA* gene (I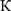 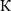
B kinase) and *RELA* (encodes the p65 subunit, forming a heterodimer with p50) by HUVEC were not affected by co-incubation of cells with PLTs (Figure 6C, D and E). Incubation of HUVEC with TNF-α significantly increased the expressions of *NFKB1* and *RELA*.

### Inhibition of the NF-κB pathway abolishes the induction of ICAM-1 and E-selectin on HUVEC by SCA PLTs

**Figure 7:**
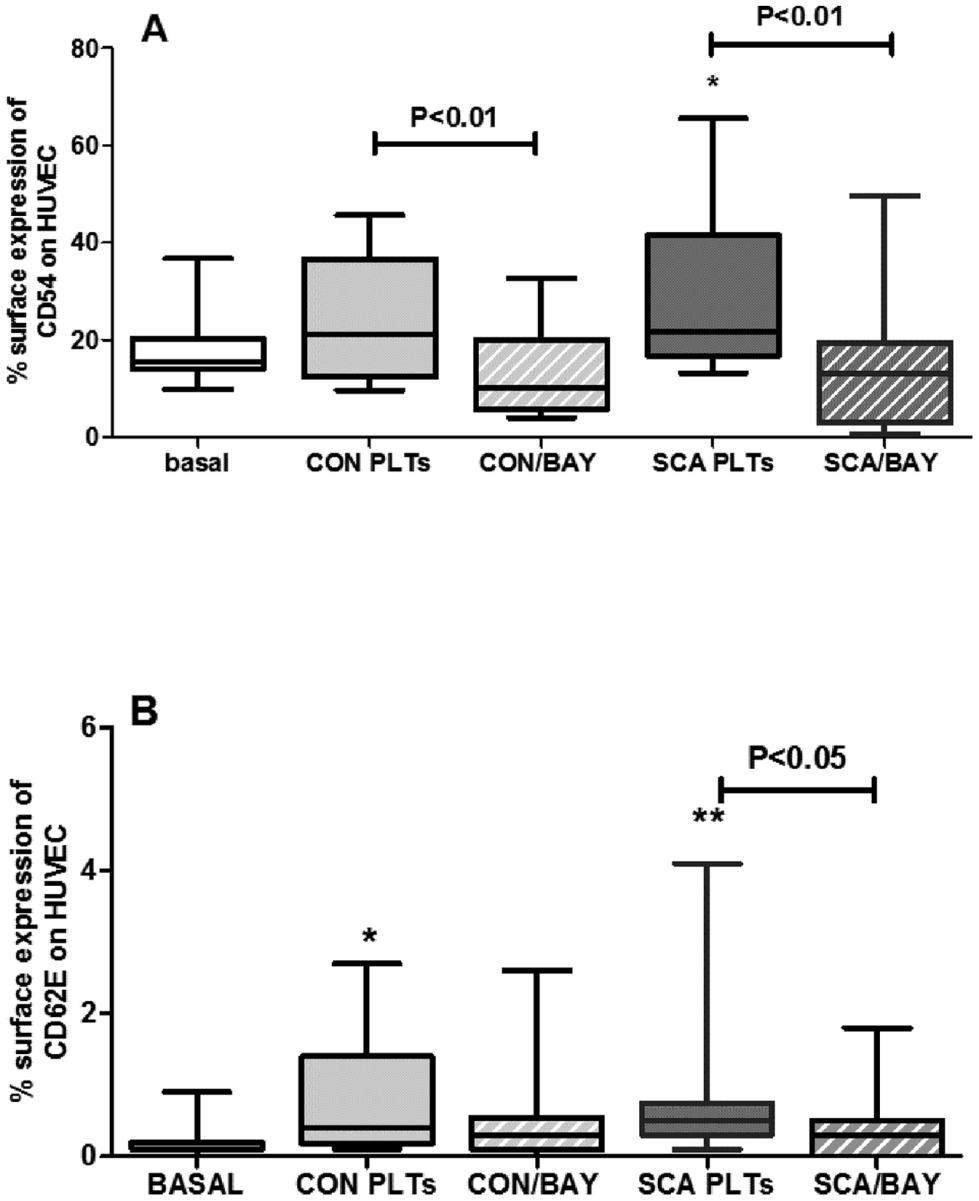
Effect of inhibition of the NF-κB pathway on the induction of endothelial adhesion molecule expression by SCA PLTs. The expressions of (A) ICAM-1 (CD54) and (B) E-selectin (CD62E) were determined by flow cytometry on HUVEC (1×10^6^ cells/well) following co-culture in direct contact with CON PLTs (N≥13) or SCA PLTs (1×10^8^ PLTs/well; N≥14) in the presence or absence of BAY 11-7082 (20 µM; 4 h, 37°C, 5% CO_2_). *, P<0.05; **, P<0.01, compared to basal (N≥12).

To determine whether the NF-κB signaling pathway mediates the SCA PLT-induced expression of adhesion molecules on the surface of HUVEC, co-cultures of HUVEC and PLTs were co-incubated with BAY 11-7082. BAY 11-7082 selectively and irreversibly inhibits phosphorylation of IκBα, resulting in a decreased activation of NF-κB [32]. BAY 11-7082 (20 µM) significantly decreased the expressions of both ICAM-1 (CD54) and E-selectin (CD62E) on the surface of HUVEC, when cells were co-cultured in the presence of SCA PLTs (FIGURE 7A). The less significant increase in ICAM-1 expression on HUVEC following incubation of cells with CON PLTs was also abolished by BAY 11-7082 (Figure 7A). Activation of HUVEC by TNF-α, as demonstrated by induction of ICAM-1 expression was also abolished by BAY 11-7082 (data not shown).

## Discussion

Previous reports consistently indicate that platelets circulate in an activated, or partially activated, state in SCD [13]. Accordingly, despite a relatively low experimental number with a limited power to detect significant differences, platelets isolated from our current cohort of patients with SCA presented a trend towards an increased expression of markers of platelet activation, including P-selectin, and an increased presentation of the α_IIb_β_3_ integrin in its activated conformation. Additionally, the production of the inflammatory cytokine, IL-1β, was augmented in SCA platelets. Previous reports on the inflammatory nature of SCA platelets indicate that SCA platelets also release increased levels of other potent cytokines such as TNFSF14 (LIGHT) and CD40L [19], as well as microparticles. In a previous study, we demonstrated an augmented α_iib_β_3_-dependent adhesion of SCA platelets, under static conditions [14]. We, herein, present data from a physiologically relevant microfluidic assay to support evidence to indicate that platelets from SCA individuals demonstrate increased adhesive properties. SCA platelets demonstrated increased adhesion to fibrinogen, compared to platelets from control individuals, strengthening our hypothesis that the adhesion of inflammatory SCA platelets to components of the blood vessel wall may be important in sickle cell disease.

Platelets from both healthy control individuals and SCA individuals were found to induce the expression of major endothelial adhesion molecules, ICAM-1 and E selectin, on the surface of HUVEC; notably the ability of SCA platelets to induce this expression was more significant and, depending on the SCA individual, levels of expression of these molecules reached levels induced by TNF-α induction. *ICAM1* gene expression by HUVEC was also induced by SCA platelets. ICAM-1 is an important ligand for leukocyte adhesion to the endothelium, while E-selectin-mediated endothelial interactions are also thought to play a role in activating leukocyte integrins, consequently increasing leukocyte adhesive properties and heterotypic interactions with other cell types [33]. Given the primary role that the adhesion of leukocytes to the vessel wall is thought to play in the vaso-occlusive process, the stimulation of endothelial-leukocyte adhesion molecule expression by activated platelets, may be important in SCD. Further evidence of the activation of the endothelium was provided by the observation that some cultures of HUVEC and SCA platelets, depending on the individual, produced higher levels of IL-8 cytokine than either HUVEC or SCA platelets alone.

Importantly, the significant effect of SCA platelets on the expression of both ICAM-1 and E-selectin on the surface of HUVEC was almost abolished by the insertion of 0.4 µm-pore transwell inserts in culture wells to separate the platelets from the endothelial cells. Data indicate that either close contact or, even, the adhesion of SCA platelets to HUVEC is required for endothelial activation by SCA platelets, rather than being an effect that is dependent just upon the release of inflammatory molecules by platelets. Platelets from SCA individuals are known to produce increased quantities of microparticles, which could play a role in endothelial activation. In addition to their pro-coagulatory properties, microparticles released from activated platelets can be internalized by endothelial cells, where they can release miR-223 and Ago2•miR-233, both able to regulate endothelial gene expression [34]. However, while platelet microparticles have been related to measure up to 1 µm in size, flow cytometry analyses indicate that microparticles released from both resting and activated platelets measure predominantly between 0.1–0.4 µm. As such, it seems likely that the 0.4 µm-transwell inserts employed herein would not prevent the majority of platelet microparticles from reaching the endothelial cells, further supporting the hypothesis that direct contact between the platelets and the endothelium induced the alterations observed, although the participation of platelet MP release cannot be ruled out.

Interestingly, SCA platelets induced the gene expression of *NFKB1*, encoding the p50 subunit of the NFκB transcription factor. Furthermore, an inhibitor of NFκB signaling, BAY 11-7082, reversed the effects of both CON and SCA platelets on the induction of ICAM-1 expression by HUVEC and inhibited the E-selection expression induced by SCA platelets. These data indicate that activation of endothelial cells by SCA platelets appears to occur via an NFκB-dependent signaling pathway. Similarly, activated monocytes from SCD individuals have been previously found to activate endothelial cell cultures in a cytokine- and NFκB-dependent manner; however, cell-to-cell contact was not required for the effect of activated monocytes on endothelial cells [35]. Such reports reinforce the multicellular nature of inflammation, endothelial activation and ensuing vaso-occlusion in SCD.

In the present study, data were acquired using platelets isolated both from steady-state SCA patients that were not in use of hydroxyurea (HU) and also from patients in use of HU. Statistical analyses revealed that the use of HU did not significantly alter the effect of SCA platelets on endothelial activation, although the relatively low experimental number employed may limit the ability to detect such differences. Moreover, in some cases, platelets from some patients that were on HU therapy activated endothelium more than platelets from some patients not on HU. While we previously [14] found that HU therapy may be associated with a reduction in platelet activation, more recent studies increasingly reveal some alterations in the current pathophysiological profile of SCA patients. HU therapy, for example, has been associated with an increase in platelet-derived plasma thrombospondin-1 in SCD patients [21] and platelet-derived TNFSF14 (LIGHT) cytokine and CD40L are not significantly altered in SCA patients on HU, compared to those not on HU [19]. Due to the ethical issues of discontinuing HU therapy in patients that fill the criteria for the use of this drug, and the transversal nature of these studies cited, it is difficult to determine whether HU therapy has no significant effect upon some aspects of platelet activation or inflammatory status, or whether, as previously suggested [21], HU therapy is now so widely used for patients with more severe SCA that, at some centers, nearly all those patients that are not in use of HU represent the patients with the mildest phenotype. Longitudinal studies would be required to establish whether HU therapy has an effect on the inflammatory status of SCA platelets.

The potential benefits of antiplatelet medications have long been discussed for SCD; earlier studies to investigate the effects of aspirin, dipyridamole and heparin, however, were inconclusive [36]–[39]. More recent ongoing trials of antiplatelet agents include those that have employed prasugrel, a P2Y(12) adenosine phosphate (ADP) receptor antagonist that inhibits platelet activation and aggregation, and eptifibatide, an α_iib_β_3_-integrin antagonist, that has also been used during acute pain episodes [40]–[43].

In conclusion, we find further evidence to indicate that platelets circulate in an activated state in sickle cell disease, demonstrating augmented inflammatory and adhesive properties. SCA platelets are capable of stimulating endothelial cell activation, as demonstrated by the induction of the increased expression of endothelial inflammatory and adhesive proteins. This effect appears to be mediated by direct contact, or even adhesion, between the platelets and endothelial cells and via an NFκB-dependent signaling pathway. In addition to the increased adhesive properties displayed by SCA platelets, it should not be forgotten that numbers of circulating platelets are generally considerably higher in SCA, as such both the quantity and activation state of platelets may contribute to endothelial activation and, therefore, the vaso-occlusive process in sickle cell disease and provide further evidence to support the use of anti-platelet approaches in association with other therapies for SCD.
